# Inflammation-Triggering Engineered Macrophages (MacTriggers) Enhance Reactivity of Immune Checkpoint Inhibitor Only in Tumor Tissues

**DOI:** 10.3390/cancers16223787

**Published:** 2024-11-10

**Authors:** Kenta Tanito, Teruki Nii, Kanae Wakuya, Yusuke Hamabe, Toma Yoshimi, Takanatsu Hosokawa, Akihiro Kishimura, Takeshi Mori, Yoshiki Katayama

**Affiliations:** 1 Graduate School of Systems Life Sciences, Kyushu University, 744 Motooka, Nishi-ku, Fukuoka 819-0395, Japan; 2 Department of Applied Chemistry, Faculty of Engineering, Kyushu University, 744 Motooka, Nishi-ku, Fukuoka 819-0395, Japan; 3 Center for Future Chemistry, Kyushu University, 744 Motooka, Nishi-ku, Fukuoka 819-0395, Japan; 4 International Research Center for Molecular Systems, Kyushu University, 744 Motooka, Nishi-ku, Fukuoka 819-0395, Japan; 5 Center for Advanced Medical Innovation, Kyushu University, 3-1-1 Maidashi, Higashi-ku, Fukuoka 812-8582, Japan; 6 Department of Biomedical Engineering, Chung Yuan Christian University, 200 Chung Pei Rd., Chung Li 32023, Taiwan

**Keywords:** macrophage, arginase 1, tumor necrosis factor-α, programmed cell death-1, immune checkpoint inhibitor, triple negative breast cancer

## Abstract

**Background:** We have previously reported engineered macrophages (MacTriggers) that can accelerate the release of tumor necrosis factor-α in response to M2 polarization. MacTriggers are characterized by two original characteristics of macrophages: (1) migration to tumors; and (2) polarization to the M2 phenotype in tumors. Intravenously administered MacTriggers efficiently accumulated in the tumors and induced tumor-specific inflammation. This study reports a novel methodology for enhancing the anti-tumor effects of immune checkpoint inhibitors (ICIs). **Results:** In this study, we newly found that the intravenously administered MacTriggers in BALB/c mouse models upregulated the expression levels of immune checkpoint proteins, such as programmed cell death (PD)-1 in CD8^+^ T cells and PD-ligand 1 (PD-L1) in cancer cells and macrophages. Consequently, in two ICI-resistant tumor-inoculated mouse models, the combined administration of MacTrigger and anti-PD-1 antibody (aPD-1) synergistically inhibited tumor growth, whereas monotherapy with aPD-1 did not exhibit anti-tumor effects. This synergistic effect was mainly from aPD-1 enhancing the tumor-attacking ability of CD8^+^ T cells, which could infiltrate into the tumors following MacTrigger treatment. Importantly, no side effects were observed in normal tissues, particularly in the liver and spleen, indicating that the MacTriggers did not enhance the aPD-1 reactivity in normal tissues. This specificity was from the MacTriggers not polarizing to the M2 phenotype in normal tissues, thereby avoiding inflammation and increased PD-1/PD-L1 expression. MacTriggers could enhance aPD-1 reactivity only in tumors following tumor-specific inflammation induction. **Conclusions:** Our findings suggest that the MacTrigger and aPD-1 combination therapy is a novel approach for potentially overcoming the current low ICI response rates while avoiding side effects.

## Simple Summary

This study proposes a novel methodology to enhance immune checkpoint inhibitor (ICI) anti-tumor effects: a combination of ICIs with inflammation-triggering engineered macrophages (MacTriggers). Intravenously administered MacTriggers significantly upregulated the expression level of immune checkpoint proteins in tumor tissues. This upregulation led to the enhancement of the anti-tumor effects of ICIs.

## 1. Introduction

Immune checkpoint inhibitors (ICIs) are one of the most commonly used type of immunotherapeutic drug for cancer therapy. ICIs can reinvigorate the anti-tumor immune response by blocking or disrupting the checkpoint protein of co-inhibitory signaling between T cells and cancer cells, such as programmed cell death (PD)-1, PD-ligand 1 (PD-L1), or cytotoxic T-lymphocyte-associated protein 4 (CTLA-4) [1,2,3]. Unlike traditional anti-cancer drugs, ICIs are designed to stimulate the patient’s own immune system, which can reduce potential side effects. While ICIs have significantly impacted cancer therapy, the current patient response rate is only approximately 10%–25% [4,5]. Although several reasons for this low ICI response rate have been reported, the primary factor is low expression levels of the target checkpoint protein in patient tumor tissues [6,7]. Considering these findings, various reports have described combining ICI therapy with scientific technologies aimed at enhancing these checkpoint protein expression levels [8,9], such as using antibody–drug conjugates (ADCs) [10]. ADCs can efficiently target antigens on the surface of cancer cells, allowing the conjugated drug to enhance checkpoint protein expression and take effect in tumor tissues. While this ADC system can achieve tumor-specific effects, its versatility is not very high because these effects may be significantly reduced if the targeted antigen is mutated or lost [11]. Additionally, this approach would not yield significant effects if the tumor does not originally express the antigen or if the expression levels are low. Moreover, if the target antigen is even slightly expressed in normal tissues, the ADC may also affect these tissues and induce side effects [12]. Other technologies can enhance checkpoint protein expression without targeting a specific antigen, including nanoparticles encapsulating drugs [13,14], microRNAs (miRNAs) [9], or genes [15]. These particles enable tumor-targeting delivery with enhanced permeation and retention (EPR) effects, a highly permeable condition of cancer blood vessels. However, it has been reported that EPR effects would be limited in stroma-rich cancers, such as pancreatic and breast cancers [16]. Recently, oncolytic adenoviruses or reoviruses have been enabled to enhance the therapeutic effects of ICIs [17,18]. For instance, combining aPD-1 with oncolytic reovirus extended the survival period in the B16 mouse melanoma model [18]. However, the risk of normal tissues being exposed to the virus remains [19]. Additionally, the encapsulation of the virus requires complicated methods [19]. Taken together, highly versatile technologies are needed that (1) can be applied regardless of the tumor type, (2) avoid affecting normal tissues, and (3) are easy to handle. Implementing these technologies could significantly improve the current low ICI patient response rates.

**Figure 1:**
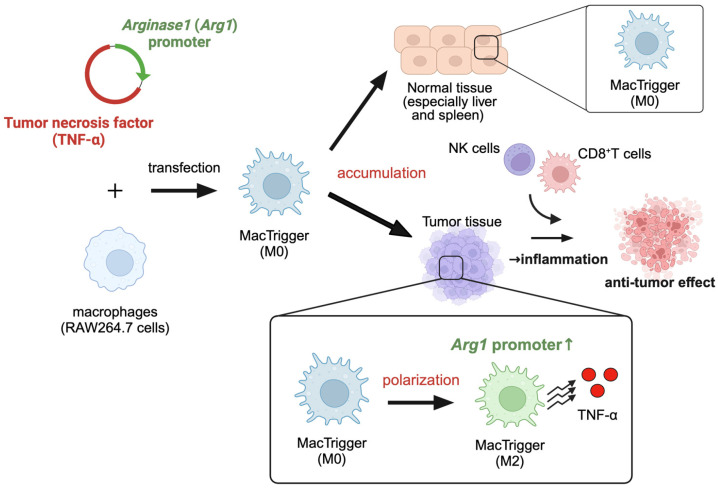
MacTriggers are engineered macrophages that express tumor necrosis factor (TNF)-α in response to *Arg1* promoter activity. MacTriggers can migrate to tumor tissues and then polarize from the M0 state to the M2 state. TNF-α secreted from MacTriggers can induce CD8^+^ T cell and natural killer (NK) cell infiltration, resulting in anti-tumor effects. In normal tissues, especially the liver and spleen, MacTriggers do not polarize from the M0 state to the M2 phenotype, nor do they express TNF-α. This figure was created with BioRender.com.

Macrophages, one of the major immune cell types, play a crucial role in controlling inflammation. Macrophages have two significant characteristics that contribute to the immunosuppressive (anti-inflammatory) microenvironment of tumor tissues [20]. The first characteristic is their efficient accumulation within tumor tissues. This is primarily attributed to chemotaxis, such as the secretion of C-C chemokine ligand 2 (CCL2) and granulocyte-macrophage colony-stimulating factor (GM-CSF) from cancer and stromal cells. Therefore, this does not depend on cancer cell antigens or EPR effects [21,22]. Second, macrophages polarize to the anti-inflammatory M2 phenotype after accumulation in tumor tissues [23,24]. M2 macrophages highly express anti-inflammatory genes (e.g., *Mrc1* and *Fizz1*) or proteins (e.g., interleukin-10 and transforming growth factor (TGF)-β) and contribute to the immunosuppressive microenvironment [20,22]. Macrophage accumulation and M2 polarization are observed irrespective of cancer type, although they may slightly differ in extent [25]. Focusing on these two unique characteristics, we recently reported modified macrophages that can promote tumor necrosis factor-α (TNF-α) release in response to arginase 1 (*Arg1*) activity [26]. *Arg1* activity is a well-known specific M2 macrophage marker. Intravenously administered engineered macrophages can migrate to the tumor tissues in accordance with the innate capacity of macrophages to accumulate within these tissues. Subsequently, the engineered macrophages can induce inflammation in the tumor tissues through the *Arg1* activity-responsive release of TNF-α. Importantly, the engineered macrophages that inadvertently accumulate in normal tissues do not undergo M2 polarization, indicating that *Arg1* activity-responsive TNF-α accelerated release does not occur, which prevents inflammation. Indeed, the damage to normal tissues was not observed. Therefore, these engineered macrophages that trigger inflammation only in tumor tissues have been named MacTriggers (Figure 1). MacTriggers are easy to prepare using lipofection of the conventional transfection methods, which only takes 3 days to complete.

In this study, we report a novel methodology aiming to enhance ICI anti-tumor effects: a combination of ICIs with MacTriggers. Here, we investigated the anti-tumor effects of this approach in 4T1 breast cancer and Colon-26 colon cancer cell lines because 4T1 and Colon-26 tumors reportedly exhibit low ICI responsiveness, and they have therefore been widely used as representative ICI-resistant cancer cell lines [27,28]. First, we assessed the elevated expression levels of PD-L1 on cancer cells and macrophages, as well as those of PD-1 on CD8^+^ T cells in tumor tissues, following MacTrigger administration in mice. Subsequently, we investigated the detailed mechanisms underlying the higher checkpoint protein expression patterns mediated by MacTriggers. After confirming this, we evaluated the anti-tumor effects of administering an anti-PD-1 antibody (aPD-1) combined with MacTrigger compared with aPD-1 or MacTrigger treatment alone. The anti-tumor effects of the combination therapy (aPD-1 with MacTrigger) were synergistically enhanced, whereas monotherapy with aPD-1 did not result in sufficient effects. In addition, significant side effects in normal tissue were not confirmed. Notably, similar synergistic effects were observed across two ICI-resistant tumor types. This study emphasizes the significant potential of MacTriggers to enhance the therapeutic efficacy of ICIs across several cancers, offering a promising avenue to overcome the current low ICI response rates.

## 2. Materials and Methods

### 2.1. Materials

Dulbecco’s modified Eagle medium (DMEM), Roswell Park Memorial Institute-1640 (RPMI-1640) medium, Antibiotic-Antimycotic Mixed Stock Solution, Penicillin–Streptomycin–Glutamine Mixed Solution, 4% paraformaldehyde phosphate buffer (4%-PFA PB), Hanks’ Balanced Salt Solution (HBSS), and Dulbecco’s phosphate-buffered saline (PBS) were purchased from Nacalai Tesque, Inc. (Kyoto, Japan). Fetal bovine serum (FBS) was purchased from Nichirei Biosciences, Inc. (Tokyo, Japan). Lipofectamine 3000 was purchased from Thermo Fisher Scientific, Inc. (Waltham, MA, USA). NucleoBond Xtra Maxi Plus EF, In-Fusion Snap Assembly Master Mix, and PrimeSTAR Max DNA polymerase were purchased from Takara Bio, Inc. (Shiga, Japan). G418 sulfate was purchased from FUJIFILM Wako Pure Chemical Corporation (Osaka, Japan). The tumor dissociation kit (mouse) was purchased from Miltenyi Biotec (Bergisch Gladbach, Germany). The brilliant violet (BV) 421-labeled anti-mouse F4/80 antibody, Alexa Fluor 488-labeled anti-mouse CD11b antibody, phycoerythrin (PE)-labeled anti-mouse PD-L1 antibody, BV421-labeled anti-mouse PD-1 antibody, PE-labeled anti-mouse T cell receptor (TCR)-β antibody, Alexa Fluor 488-labeled anti-mouse CD8a antibody, allophycocyanin (APC)-labeled anti-mouse CD4 antibody, BV421-labeled anti-mouse forkhead box P3 (Foxp3) antibody, BV421-labeled anti-mouse CD45 antibody, PE-labeled anti-mouse lymphocyte antigen 6 family member G (Ly6G) antibody, PE-labeled anti-mouse TNF-α antibody, APC-labeled anti-mouse interferon-gamma (IFN-γ) antibody, APC-labeled anti-mouse TNF-α antibody, PE-labeled anti-mouse CD206 antibody, Enzyme-Linked Immunosorbent Assay (ELISA) MAX™ Deluxe Set Mouse TNF-α, Zombie NIR™ Fixable Viability Kit, True-Nuclear™ Transcription Factor Buffer Set, Brefeldin A, and stop solution for TMB substrate were purchased from BioLegend, Inc. (San Diego, CA, USA). The eBioscience™ Intracellular Fixation & Permeabilization Buffer Set was purchased from Invitrogen (Carlsbad, CA, USA). The Ki67 (D3B5) Rabbit mAb was purchased from Cell Signaling Technology, Inc. (Danvers, MA, USA). Horseradish peroxidase (HRP)-labeled rabbit antibody and 3, 3-Diaminobenzidine (DAB) were purchased from Dako, Inc. (Glostrup, Denmark). The InVivoMAb anti-mouse PD-1 antibody (aPD-1) was purchased from BioXcell (Lebanon, NH, USA). Phorbol 12-Myristate 13-Acetat (PMA) and Ionomycin calcium salt from *Streptomyces conglobatus* were purchased from Sigma-Aldrich (St. Louis, MO, USA). IVISense 680 Fluorescent Cell Labeling Dye (Vivo-Track 680) was purchased from PerkinElmer, Inc. (Waltham, MA, USA). VersaLyse was purchased from Beckman Coulter, Inc. (Brea, CA, USA). Cyclophosphamide monohydrate and doxorubicin hydrochloride were purchased from Tokyo Chemical Industry Co., Ltd. (Tokyo, Japan). All other chemicals were of the highest grade commercially available.

### 2.2. Cell Culture

RAW264.7 murine macrophages and 4T1 murine breast cancer cells were purchased from the American Type Culture Collection (ATCC; Manassas, VA, USA). Colon-26 murine colon cancer cells were purchased from the RIKEN BRC cell bank (Ibaraki, Japan). RAW264.7 cells were cultured in DMEM. 4T1 cells and Colon-26 cells were cultured in RPMI-1640. The RAW264.7 and Colon-26 cell culture media were supplemented with 10% heat-inactivated FBS and 1% Antibiotic-Antimycotic Mixed Stock Solution. The 4T1 cell culture medium was supplemented with 10% heat-inactivated FBS and 1% Penicillin–Streptomycin–Glutamine Mixed Solution. Cells were cultured in an atmosphere containing 5% CO_2_ and 95% air at 37 °C.

### 2.3. Evaluation of Macrophage Characteristics in 4T1 and Colon-26 Tumor-Bearing Mice

All animal experiments were performed with approval from the Institutional Animal Care and Use Committee of Kyushu University. Female BALB/cAJcl mice aged 5–7 weeks were obtained from Kyudo Japan (Saga, Japan). We first aimed to confirm the two macrophage functions: (1) accumulation within the tumor tissues and (2) M2 polarization in the tumor tissues. After a week of breeding, 4T1 cells or Colon-26 cells (2 × 10^5^ cells in 50 μL HBSS) were subcutaneously (s.c.) injected into the back. Five days after inoculation, Vivo-Track 680 (VT-680)-labeled RAW264.7 macrophages (1 × 10^6^ cells in 100 μL PBS) were administered via tail vein injection. Then, 1 day and 4 days after macrophage administration, several tissues (tumor, liver, spleen, kidney, and lung) were extracted. After extraction, macrophage accumulation was evaluated using the IVIS Spectrum (IVIS Lumina II, Xenogen Co., Alameda, CA, USA). The tumor, liver, and spleen tissues were then washed with PBS. The tumors were digested using a tumor dissociation kit according to the manufacturer’s protocol. Then, the cells were filtered through a 100 μm filter and centrifuged for 5 min at 500× *g*. The liver and spleen tissues were homogenized using a plunger on a 100 μm filter. Next, the cells were suspended in FACS buffer (PBS containing 2% FBS). After centrifugation, the cells were treated with VersaLyse to lyse red blood cells (RBCs) and incubated for 5 min at room temperature, after which the cells were washed with FACS buffer. Then, several fluorescent-labeled antibodies (BV421-labeled anti-mouse F4/80 antibody, Alexa Fluor 488-labeled anti-mouse CD11b antibody, and PE-labeled anti-mouse CD206 antibody) were added and incubated for 30 min at 4 °C. Next, the cells were washed twice and resuspended in FACS buffer. The CD206 expression levels of administered macrophages (F4/80^+^, CD11b^+^, and VT-680^+^) were analyzed by flow cytometry (CytoFLEX, Beckman Coulter Inc., Brea, CA, USA). The gating and analysis were performed using FlowJo software.

### 2.4. MacTrigger Production

MacTriggers were prepared by transfection according to the previously reported method [26]. Briefly, plasmid DNA expressing TNF-α in response to the *Arg1* promoter was prepared by an In-fusion method. Plasmid DNA extraction was conducted using NucleoBond Xtra Maxi Plus EF according to the manufacturer’s protocol. Next, RAW264.7 cells were seeded into 24-well plates at a density of 2.0 × 10^5^ cells/well. After 24 h, the plasmid DNA was transfected into the RAW264.7 cells using Lipofectamine 3000 reagent according to the manufacturer’s protocol. Six hours after transfection, the medium was replaced with fresh medium. The cells were treated with 400 μg/mL G418 to obtain stable cell lines. After the selection, single-cell cloning was performed according to the general limiting dilution protocol. TNF-α expression was confirmed by ELISA MAX™ Deluxe Set Mouse TNF-α according to the manufacturer’s protocol. We named these engineered macrophages MacTriggers.

### 2.5. Enhanced Green Fluorescent Protein (EGFP)-Expressing Cancer Cell Preparation

EGFP-expressing 4T1 cells and Colon-26 cells were prepared using Lipofectamine 3000 reagent according to the general protocol. EGFP-expressing 4T1 cells and Colon-26 cells were obtained using the same methods as described in Section 2.4. EGFP expression was analyzed by flow cytometry.

### 2.6. Effects of MacTriggers on Checkpoint Protein Expression in Tumor and Normal Tissues

The two types of tumor-bearing mice (4T1 and Colon-26) were obtained using the same methods as described in Section 2.3. EGFP-expressing 4T1 cells or Colon-26 cells were used when evaluating PD-L1 expression in cancer cells. Five days after tumor inoculation, MacTrigger (1 × 10^6^ cells in 100 μL PBS) was administered via a tail vein injection. Then, 4 or 8 days after MacTrigger administration, the tumors were extracted and digested using a tumor dissociation kit according to the manufacturer’s protocol. The cells were filtered through a 100 μm filter and centrifuged for 5 min at 500× *g*, then resuspended in PBS and incubated with the viability dye (Zombie-NIR) for 15 min at 4 °C. After the incubation, the cells were washed twice and resuspended in FACS buffer. The extracted liver and spleen tissues were homogenized using a plunger on a 100 μm filter. Following collection, the cells were treated with VersaLyse to lyse RBCs and incubated for 5 min at room temperature. Next, the cells were washed with FACS buffer. Then, several fluorescent-labeled antibodies (BV421-labeled anti-mouse PD-1 antibody, PE-labeled anti-mouse TCR-β antibody, Alexa Fluor 488-labeled anti-mouse CD8a antibody, BV421-labeled anti-mouse F4/80 antibody, Alexa Fluor 488-labeled anti-mouse CD11b antibody, BV421-labeled anti-mouse CD45 antibody, and PE-labeled anti-mouse PD-L1 antibody) were added and incubated for 30 min at 4 °C. After the incubation, the cells were washed twice and resuspended in FACS buffer. The expression levels of PD-L1 in macrophages (F4/80^+^, CD11b^+^, and PD-L1^+^), PD-L1 in cancer cells (CD45^−^, EGFP^+^, and PD-L1^+^), and PD-1 in CD8^+^ T cells (TCR-β^+^, CD8^+^, and PD-1^+^) were analyzed by flow cytometry. The gating and analysis were performed using FlowJo software. The gating strategy is shown in Figure S1.

### 2.7. Evaluation of Immune Cell Infiltration in Tumor Tissues

To investigate the mechanisms by which MacTrigger can enhance immune checkpoint protein expression levels, we evaluated immune cell infiltration in tumor tissues. The tumor inoculation, MacTrigger administration, and tumor dissociation procedures were performed as described in Section 2.6. The collected cells from tumor tissues were incubated with the viability dye (Zombie-NIR) for 15 min at 4 °C. The cells were then resuspended in FACS buffer, and the fluorescent-labeled antibodies (PE-labeled anti-mouse TCR-β antibody and APC-labeled anti-mouse CD4 antibody) were added. Then, the cells were washed twice and resuspended in FACS buffer. Next, for intracellular factor staining, the cells were treated with a True-Nuclear™ Transcription Factor Buffer Set according to the manufacturer’s protocol. After the fixation and permeabilization, Foxp3 was stained using a BV421-labeled anti-mouse Foxp3 antibody. Finally, regulatory T cell (Treg) (TCR-β^+^, CD4^+^, and Foxp3^+^) infiltration was analyzed by flow cytometry. The gating strategy is shown in Figure S1.

### 2.8. Evaluation of Intracellular Cytokine Expression

To further investigate the mechanism, intracellular cytokine expression patterns were evaluated. The tumor inoculation, MacTrigger administration, and tumor dissociation procedures were performed as described in Section 2.6 and 2.7. The tumor-infiltrating cells were collected and incubated with activation buffer (RPMI-1640 containing 10% FBS, 50 ng/mL PMA, 250 ng/mL Ionomycin, and Brefeldin A) for 4.5 h at 37 °C. The cells were centrifuged for 5 min at 500× *g*, and then the supernatant was removed. Next, the viability dye (Zombie-NIR) was added and incubated for 15 min at 4 °C. The cells were washed twice and resuspended in FACS buffer. Subsequently, the fluorescent-labeled antibodies (BV421-labeled anti-mouse CD45 antibody, Alexa Fluor 488-labeled anti-mouse CD11b antibody, PE-labeled anti-mouse Ly6G antibody, BV421-labeled anti-mouse F4/80 antibody, or PE-labeled TCR-β antibody) were added, respectively, and incubated for 30 min at 4 °C. Then, the cells were washed twice and treated with the eBioscience™ Intracellular Fixation & Permeabilization Buffer Set according to the manufacturer’s protocol. After the fixation and permeabilization, the fluorescent-labeled antibodies (PE-labeled anti-mouse TNF-α antibody, APC-labeled anti-mouse IFN-γ antibody, or APC-labeled anti-mouse TNF-α antibody) were added for intracellular cytokine staining and incubated for 30 min at 4 °C. Then, the cells were washed twice and resuspended in FACS buffer. The TNF-α and IFN-γ expression levels in macrophages (F4/80^+^ and CD11b^+^), T cells (CD45^+^ and TCR-β^+^), and neutrophils (CD45^+^, CD11b^+^, and Ly6G^+^) were analyzed by flow cytometry. The gating strategy is shown in Figure S2.

### 2.9. Evaluation of Anti-Tumor Effects in Tumor-Bearing Mice

The anti-tumor effects of aPD-1 combined with MacTrigger were evaluated. The two types of tumor-bearing mice (4T1 and Colon-26) were obtained as described in Section 2.6,Section 2.7,Section 2.8. Five days after tumor inoculation, the mice were randomized in four groups. PBS (100 μL), aPD-1 (50 μg in 100 μL PBS), MacTrigger (1 × 10^6^ cells in 100 μL PBS), or MacTrigger+aPD-1 (1 × 10^6^ cells and 50 μg in 100 μL PBS) was intravenously administered. Tumor volumes and body weights were measured every 2 days. The tumor volumes were calculated using the following formula.

Tumor volume = 0.5 × Major axis × (Minor axis)^2^ (1)

When the tumor major axis reached 20 mm (humane endpoint), the experiment was finished, and the mice were sacrificed. The tumor, liver, and spleen tissues were then extracted. The liver and spleen tissue weights were measured to assess the tissue enlargement associated with injury.

### 2.10. Histological Analysis

To visually investigate the effects on normal tissues, hematoxylin and eosin (H&E) staining was performed according to the general protocol. In addition, immunohistochemistry (IHC) was performed according to the general protocol to investigate the proliferation of cancer cells. Fourteen days after treatment, several tissues (tumor, liver, spleen, kidney, and lung) were extracted and fixed in 4%-PFA PB. For H&E staining, several tissues were deparaffinized and washed with water. Hematoxylin was added and incubated for 5 min. Then, the slides were washed and hydrated. Eosin was added and incubated for 5 min. After the incubation, the slides were washed. For IHC, sections embedded in paraffin blocks were used for Ki67 staining. Tumor tissues were deparaffinized, hydrated, and heated in citric acid buffer (pH 6.0) to 95 °C for 15 min, then blocked with 3% skim milk for 10 min. The slides were incubated overnight with an anti-Ki67 antibody (1:200). Then, the slides were washed with PBS and incubated with 0.3% hydrogen peroxide–methanol buffer for 30 min to block endogenous peroxidase activity. After washing with PBS, HRP-labeled secondary antibody was added and incubated for 30 min. The slides were then washed with PBS, and DAB was added as an enzyme substrate. All of the H&E and IHC section images were observed using a fluorescent microscope (BZ-X800, KEYENCE, Osaka, Japan).

### 2.11. Statistical Analysis

GraphPad Prism software (GraphPad software, San Diego, CA, USA) was used for all statistical analysis procedures. For a normal distribution, a two-way analysis of variance (ANOVA) followed by Tukey’s post hoc test was used for multiple comparisons, and a two-tailed Welch’s t-test was used for single comparisons. For a non-normal distribution, the Kruskal–Wallis test was used for multiple comparisons, and the Mann–Whitney test was used for single comparisons. All data are indicated as medians with interquartile ranges (IQRs). The symbols * and ** indicate *p*-values less than 0.05 and 0.01, respectively, while ns indicates no significant differences.

## 3. Results and Discussion

### 3.1. Evaluation of Immune Checkpoint Protein Expression and the Underlying Mechanism

**Figure 2:**
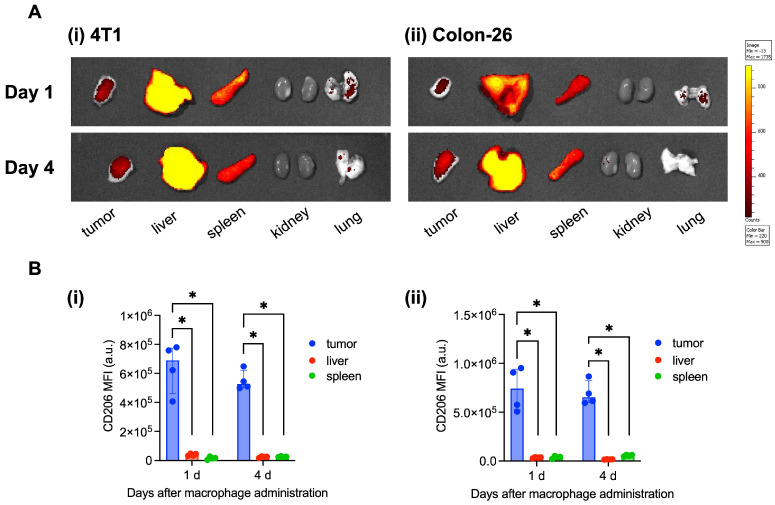
(**A**) Ex vivo imaging of administered RAW264.7 macrophage accumulation in several tissues (tumor, liver, spleen, kidney, and lung) 1 and 4 days after administration in 4T1 (**i**) and Colon-26 (**ii**) tumor-bearing mice. (**B**) Mean fluorescent intensity of CD206 of administered macrophages in tumor, liver, and spleen tissues 1 and 4 days after macrophage administration in 4T1 (**i**) and Colon-26 (**ii**) tumor-bearing mice (*n* = 4, median (IQR)). * *p* < 0.05.

First, we confirmed the two macrophage characteristics: (1) accumulation within the tumor tissues and (2) tumor-specific M2 polarization in tumors originating from 4T1 and Colon-26 cells. The macrophages migrated to the tumor, liver, and spleen tissues 1 and 4 days after intravenous administration (Figure 2A). In addition, M2 polarization (upregulation of CD206 expression) was observed only with the macrophages that had accumulated within tumor tissues (Figure 2B). Therefore, we confirmed macrophage accumulation and M2 polarization in both 4T1 and Colon-26 tumor-bearing mice, which strongly supports our previous studies [26].

**Figure 3:**
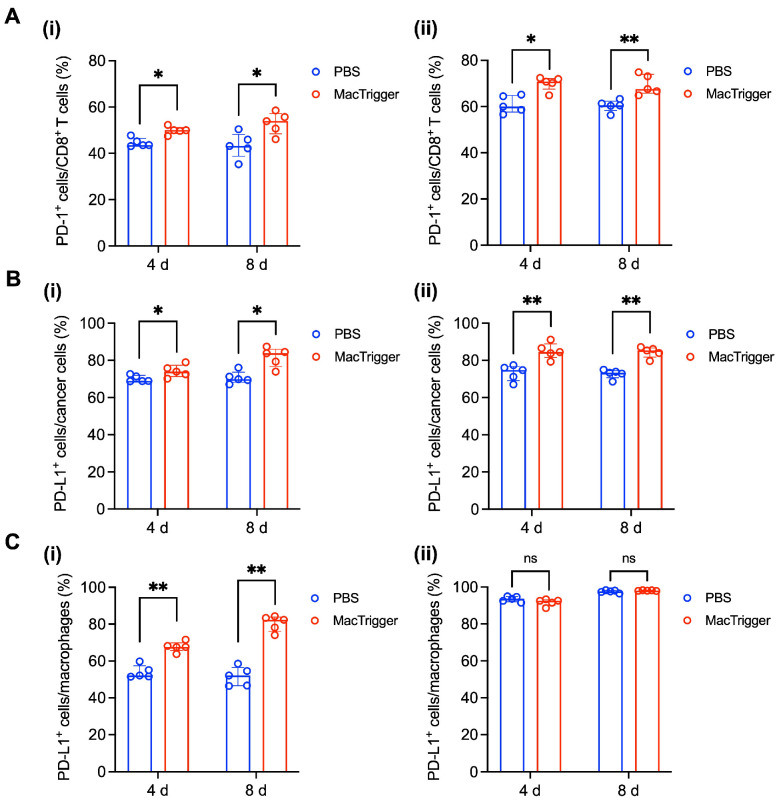
(**A**) Percent of programmed cell death (PD)-1^+^ cells in CD8^+^ T cells 4 and 8 days after MacTrigger administration in 4T1 (**i**) and Colon-26 (**ii**) tumors (*n* = 5, median (IQR)). (**B**) Percent of PD-ligand 1 (PD-L1)^+^ cells in cancer cells 4 and 8 days after MacTrigger administration in 4T1 (**i**) and Colon-26 (**ii**) tumors (*n* = 5, median (IQR)). (**C**) Percent of PD-L1^+^ cells in macrophages 4 and 8 days after MacTrigger administration in 4T1 (**i**) and Colon-26 (**ii**) tumors (*n* = 5, median (IQR)). * *p* < 0.05, ** *p* < 0.01. ns: not significant.

Next, we investigated the effects of MacTrigger administration on the tumor tissues. First, the PD-1 and PD-L1 immune checkpoint protein expression levels were evaluated. As shown in Figure 3A,B, similar trends of upregulated PD-1 (CD8^+^ T cells) and PD-L1 (cancer cells) expression levels were observed between 4T1 and Colon-26 tumor-bearing mice 4 and 8 days following MacTrigger administration. However, differences were found between the two tumor types for PD-L1 expression on macrophages (Figure 3C). Upregulated PD-L1 expression in macrophages was not observed in Colon-26 tumor-bearing mice, likely because of the innate expression level of PD-L1. Figure 3C(ii) indicates that more than 90% of PD-L1^+^ macrophages were originally present in the Colon-26 tumors. Therefore, it is conceivable that PD-L1 expression was already saturated by several cytokines in the tumor tissues [29,30], causing MacTrigger-mediated PD-L1 upregulation to not be observed in the macrophages. For instance, the importance of IL-6 in PD-L1 expression has been recently noted, especially in colon tumors [31]. IL-6 promotes the expression of PD-L1 on macrophages via JAK/STAT signaling, and the expression of PD-L1 on macrophages is more durable than PD-L1 in cancer cells [32]. Considering these reports, it is conceivable that the expression of PD-L1 in macrophages would tend to be saturated in Colon-26 tumors. Although the upregulated levels differed from tumor to tumor because of the original expression patterns, these findings suggest that MacTrigger could enhance immune checkpoint protein expression in tumor tissues.

**Figure 4:**
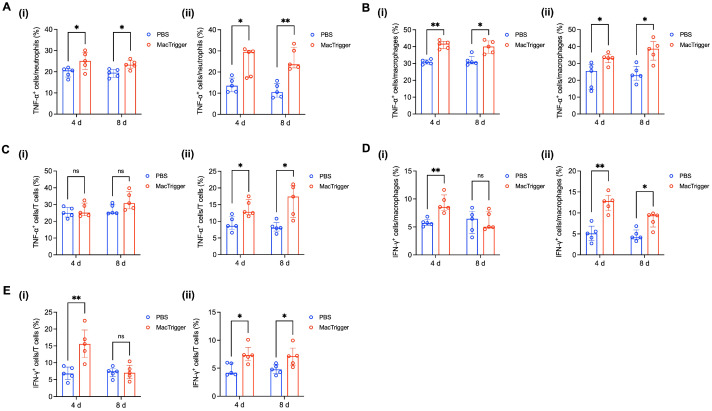
(**A**) Percent of tumor necrosis factor (TNF)-α^+^ cells in neutrophils 4 and 8 days after MacTrigger administration in 4T1 (**i**) and Colon-26 (**ii**) tumors (*n* = 5, median (IQR)). (**B**) Percent of TNF-α^+^ cells in macrophages 4 and 8 days after MacTrigger administration in 4T1 (**i**) and Colon-26 (**ii**) tumors (*n* = 5, median (IQR)). (**C**) Percent of TNF-α^+^ cells in T cells 4 and 8 days after MacTrigger administration in 4T1 (**i**) and Colon-26 (**ii**) tumors (*n* = 5, median (IQR)). (**D**) Percent of interferon (IFN)-γ^+^ cells in macrophages 4 and 8 days after MacTrigger administration in 4T1 (**i**) and Colon-26 (**ii**) tumors (*n* = 5, median (IQR)). (**E**) Percent of IFN-γ^+^ cells in T cells 4 and 8 days after MacTrigger administration in 4T1 (**i**) and Colon-26 (**ii**) tumors (*n* = 5, median (IQR)). * *p* < 0.05, ** *p* < 0.01. ns: not significant.

Next, we aimed to determine the mechanisms by which MacTrigger could enhance PD-1/PD-L1 expression. The higher PD-1 expression levels potentially resulted from CD8^+^ T cell infiltration into the tumor tissues, as our previous report demonstrated that MacTriggers can induce infiltration of this cell type [26]. The infiltrated CD8^+^ T cells are expected to express PD-1 because reports have shown that activated CD8^+^ T cells constantly express this protein [9,33,34]. To determine if a significant factor induces PD-L1 upregulated expression in cancer cells and macrophages, we assessed the TNF-α and IFN-γ expression levels in several immune cell types in the tumor tissues. Several studies have shown that Th1 cytokines, such as TNF-α and IFN-γ, secreted from tissues can enable PD-L1 expression enhancement on cancer cells or endogenous macrophages through the JAK/STAT and NF-κB signaling pathways [9,35,36]. Higher TNF-α expression levels were observed in neutrophils and macrophages in both 4T1 and Colon-26 tumors 4 and 8 days after MacTrigger administration (Figure 4A,B). In contrast, TNF-α expression from T cells was increased in Colon-26 tumors (Figure 4C). Moreover, IFN-γ expression from T cells and macrophages was increased in both 4T1 and Colon-26 tumors 4 days after MacTrigger administration (Figure 4D,E). Although there were slight differences between 4T1 and Colon-26 tumors, these data suggested that MacTriggers could enhance PD-L1 expression in cancer cells and macrophages via high expression of TNF-α and IFN-γ in several immune cell types. The reason why IFN-γ upregulation was not maintained in the 4T1 tumors is potentially because of the high level of immunosuppression [17,37,38]. Although there was a slight difference, MacTriggers conclusively enhanced the expression of Th1 cytokines in several immune cell types, resulting in the enhancement of PD-L1 expression in the tumor tissues.

Our results suggested that the conceivable mechanism of MacTrigger-mediated PD-1 and PD-L1 expression enhancement first involves triggering inflammation in the tumor tissues by accelerated TNF-α release. The TNF-α from the MacTrigger then prompts several immune cell types, such as neutrophils, T cells, and macrophages, to highly express TNF-α or IFN-γ, leading to increased PD-L1 expression in cancer cells and macrophages. CD8^+^ T cells can then efficiently migrate to the inflamed tumor tissues, resulting in higher PD-1 expression in CD8^+^ T cells within the tumor tissues. While MacTrigger can prompt the CD8^+^ T cells to attack the tumor tissues, cancer cells could likely protect themselves through the PD-1/PD-L1 interaction and weaken the CD8^+^ T cell attack efficiency. In addition, PD-L1 on macrophages can inhibit the CD8^+^ T cell attacking ability [39]. The advantage of MacTriggers is that their effects do not depend on the tumor type because the mechanisms enhancing PD-1/PD-L1 levels do not rely on antigens.

### 3.2. Anti-Tumor Effects of the aPD-1 and MacTrigger Combination Treatment in Tumor-Bearing Mice

**Figure 5:**
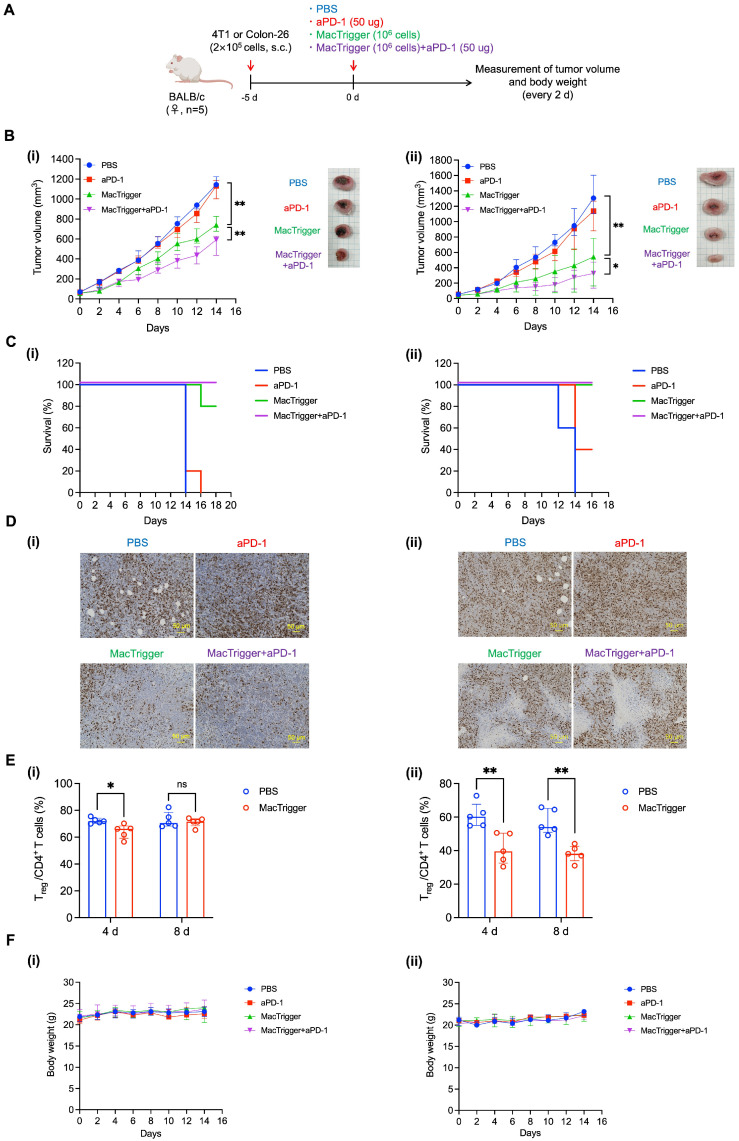
(**A**) Schematic showing the mouse experimental protocols we performed. (**B**) Time course of tumor volume measurements over 14 days in 4T1 (**i**) and Colon-26 (**ii**) tumor-bearing mice (*n* = 5, median (IQR)) and images of tumor tissues 14 days after treatment. (**C**) Survival rates of mice in each treatment group (*n* = 5). (**D**) Immunohistochemistry (IHC) staining of tumor sections excised from 4T1 (**i**) and Colon-26 (**ii**) tumor-bearing mice for Ki67 14 days after administration. Images were obtained under a microscope using a 20× objective lens. Scale bar, 50 μm. (**E**) Percent of regulatory T cells in CD4^+^ T cells 4 and 8 days after MacTrigger administration in 4T1 (**i**) and Colon-26 (**ii**) tumor-bearing mice (*n* = 5, median (IQR)). (**F**) Time course of body weight measurements in 4T1 (**i**) and Colon-26 (**ii**) tumor-bearing mice (*n* = 5, median (IQR)). * *p* < 0.05, ** *p* < 0.01. ns: not significant.

After observing upregulated PD-1/PD-L1 expression patterns following MacTrigger administration, we next assessed the anti-tumor effects of the aPD-1 and MacTrigger combination treatment. PBS (100 μL), aPD-1 (50 μg in 100 μL of PBS), MacTrigger (1 × 10^6^ cells in 100 μL of PBS), or MacTrigger + aPD-1 (1 × 10^6^ cells and 50 μg in 100 μL of PBS) was administered via the tail vein to 4T1 and Colon-26 tumor-bearing mice. The tumor volumes and body weights were measured every 2 days (Figure 5A). For this experiment, 1 × 10^6^ MacTrigger cells were selected because we determined this cell number to be suitable in our previous study [26]. In addition, a 50 μg dose of aPD-1 was used because of its frequent previous use [40,41,42]. As shown in Figure 5B, aPD-1 monotherapy did not sufficiently suppress tumor growth in 4T1 (i) or Colon-26 (ii) tumor-bearing mice. However, when aPD-1 was combined with MacTrigger, the suppression effects synergistically improved. In addition, the combination therapy group showed an extended survival period (Figure 5C) and a decreased number of Ki67-positive cells (Figure 5D). It is conceivable that the tumor-attacking ability of CD8^+^ T cells infiltrated by MacTrigger had been weakened by the cancer cells and macrophages via the PD-1/PD-L1 interaction (Figure 3A). Treatment with aPD-1 was able to restore the original ability of the CD8^+^ T cells, resulting in synergistic anti-tumor effects. Although the MacTrigger could enable aPD-1 to enhance the anti-tumor effects in these two tumor types, the degree of suppression differed. In the 4T1 tumors, the anti-tumor effects were weak compared with the Colon-26 tumors. This difference may be from the original immunosuppression level of the tumor tissues. Studies show that 4T1 tumors are severely immunocompromised [17,37,38]. Indeed, the Treg ratio, the immunosuppressive level index, was much higher in 4T1 tumors (approximately 70%) than in Colon-26 tumors and did not display a significant decrease 8 days after MacTrigger administration (Figure 5E(i)). In addition, 4T1 tumors express higher levels of immunosuppressive molecules such as TGF-β1, myeloperoxidase, and vascular endothelial growth factor than Colon-26 tumor [43]. These molecules suppress the anti-tumor immune cells and activate the anti-inflammatory immune cells (e.g., Treg, tumor-associated macrophages, and cancer-associated fibroblasts) [22,44,45,46,47]. The high level of immunosuppression associated with these tumor tissues potentially resulted in insufficient MacTrigger-induced inflammation. Body weight loss was not observed in any group (Figure 5F).

**Figure 6:**
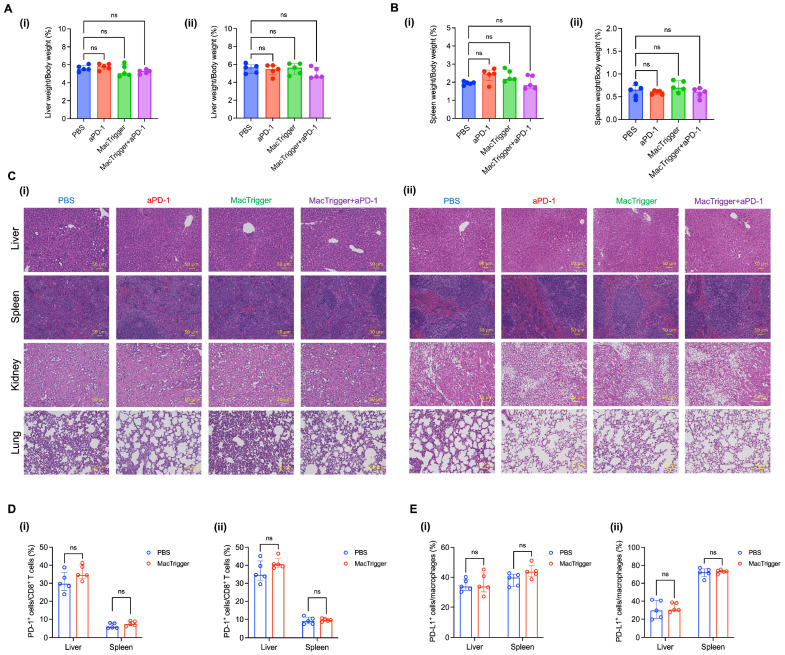
(**A**) Percent of liver weight/body weight in 4T1 (**i**) and Colon-26 (**ii**) tumor-bearing mice (*n* = 5, median (IQR)). (**B**) Percent of spleen weight/body weight in 4T1 (**i**) and Colon-26 (**ii**) tumor-bearing mice (*n* = 5, median (IQR)). (**C**) Hematoxylin and eosin (H&E)-stained tissue sections (liver, spleen, kidney, and lung) excised from 4T1 (**i**) and Colon-26 (**ii**) tumor-bearing mice 14 days after treatment. Images were obtained under a microscope using a 20× objective lens. Scale bar, 50 μm. (**D**) Percent of programmed cell death (PD)-1^+^ cells in CD8^+^ T cells in liver and spleen tissues 4 days after MacTrigger administration in 4T1 (**i**) and Colon-26 (**ii**) tumors (*n* = 5, median (IQR)). (**E**) Percent of PD-ligand 1 (PD-L1)^+^ cells in macrophages in liver and spleen tissues 4 days after MacTrigger administration in 4T1 (**i**) and Colon-26 (**ii**) tumors (*n* = 5, median (IQR)). ns: not significant.

To assess potential adverse effects, the liver and spleen weights were measured because macrophages tend to accumulate in these two organs (Figure 2A). In all groups, no injury-derived tissue enlargement was observed (Figure 6A,B). To further investigate potential tissue injury in more detail, we performed H&E staining of the liver, spleen, kidney, and lung tissue samples. No inflammation-related immune cell infiltration was observed, including in the combination therapy group (Figure 6C). This was because the MacTrigger did not polarize to the M2 phenotype (Figure 2B) or trigger inflammation in the normal tissues, resulting in no effects to the PD-1/PD-L1 expression patterns (Figure 6D,E). In this case, the aPD-1 reactivity would not change before or after MacTrigger administration. This emphasizes the advantages of using MacTrigger: the normal tissues remain unaffected with no ICI-derived immune-related adverse events (irAEs), which is a concern associated with current ICI therapy. When an ICI is combined with MacTrigger administration, the ICI anti-tumor effects could be enhanced without inducing irAEs.

In this study, we selected the simultaneous administration of aPD-1 and MacTrigger after investigating the impacts of the treatment order on the anti-tumor and adverse effects in 4T1 tumor-bearing mice (Figure S3). We examined three groups: simultaneous administration (group 1), MacTrigger administration first (group 2), and aPD-1 administration first (group 3). Tumor growth was not sufficiently suppressed in group 3 because MacTrigger-derived PD-1/PD-L1 upregulation was not induced prior to antibody treatment. On the other hand, the anti-tumor effects improved in both groups 1 and 2 (Figure S4). No significant difference in anti-tumor effects (Figure S4), survival period (Figure S5), or transition of body weight (Figure S6) was observed between the two groups, likely because of the blood half-life of aPD-1. In a previous report, aPD-1 accumulation in the tumor tissues was retained 7 days after administration [48]. Therefore, it is conceivable that the administered aPD-1 would continue to be present and affect the tumor tissues while the MacTrigger enhanced PD-1/PD-L1 expression. For side effects, no liver or spleen enlargement was seen in any group (Figures S7 and S8). Taken together, the treatment schemes explored in groups 1 and 2 showed no clear difference in results. Considering its simplicity, simultaneous administration of MacTrigger and aPD-1 was chosen as the appropriate approach. Moreover, the data shown in Figure 3A–C indicated that the multiple administration of aPD-1 could enhance the therapeutic effect of the combination therapy because PD-1 and PD-L1 expression upregulation was retained 8 days after MacTrigger administration. Therefore, we aimed to evaluate the anti-tumor effects of the combination therapy with aPD-1 multiple administration on days 0, 4, and 8 (Figure S9). As shown in Figure S10, repeated administration of aPD-1 did not result in drastic improvements compared with single administration in both 4T1 (i) and Colon-26 (ii) tumor-bearing mice. This was especially true in 4T1 tumors, likely from the high immunosuppression. The effects of MacTrigger, such as decreased levels of Tregs and inflammatory cytokine expression, might be counteracted by the original immunosuppressive level of the 4T1 tumors. Further investigation is needed to explore why this system did not result in the full disappearance of these tumor tissues.

The 4T1 tumor-bearing mice are widely used as triple-negative breast cancer (TNBC) models [38,49], and therefore, this study also provides potential new therapeutic options for this disease. TNBC is a clinically aggressive breast cancer subtype that accounts for approximately 15% of breast cancer cases [50,51]. The poor patient outcomes associated with TNBC are mainly from (1) very few target proteins and (2) high levels of immunosuppression [17,37,38]. As the name suggests, TNBC tumors lack expression of the three common therapeutic targets: estrogen receptor, progesterone receptor, and human epidermal growth factor receptor 2 [52]. Because of these characteristics, the therapeutic options for TNBC are limited, with the disease typically being managed using standard chemotherapy drugs, such as doxorubicin (DOX), cyclophosphamide (CPA), or paclitaxel [53,54]. However, these anti-cancer drugs can also affect normal tissues, resulting in severe side effects, such as a drastic decrease in body weight or hair loss. Indeed, despite using the standard DOX and CPA doses for TNBC, the body weight of the mice in this study reached a decrease of 20% (humane endpoint) after only 8 days of treatment (Figure S11). Because the induced inflammation and PD-1/PD-L1 upregulation associated with MacTrigger treatment do not depend on cancer antigens, the MacTrigger and aPD-1 combination showed therapeutic efficacy for TNBC. The high level of immunosuppression, the second reason for the poor TNBC therapy outcomes, signifies the low expression of PD-1 and PD-L1 that ICI can react to [55]. This suggests that immunotherapies, such as aPD-1 or anti-PD-L1 (aPD-L1), exhibit low therapeutic effects, as shown in Figure 5B(i). Although aPD-1 and aPD-L1 have recently been clinically approved for treating TNBC, many reports still indicate poor outcomes [56,57,58]. Because the MacTrigger-induced inflammation is triggered by immunosuppressive genes (*Arg1*), MacTrigger can also work in tumors with high levels of immunosuppression. Therefore, the two characteristics of MacTrigger (antigen independence and triggered by immunosuppression) work well for TNBC. The MacTrigger and aPD-1 combination therapy is therefore promising as a new TNBC therapeutic option.

On the other hand, there are limitations to the application to humans. First, the *Arg1* promoter cannot be used in humanized MacTriggers because the expression of *Arg1* is low in human M2 macrophages [59]. Therefore, the M2-specific promoter of human macrophages should be screened. Second, the actual tumor environment of humans is more complicated than mouse tumor models [60]. Therefore, a humanized mouse model is needed to investigate the therapeutic effect and immune response in humanized MacTriggers further. Moreover, the complicated environment of the tumor may affect the biological functions of TNF-α and decrease the therapeutic effects of MacTrigger. Therefore, biological functions such as binding capacity and conformation stability should be evaluated in tumor mimic conditions. Although there remains room for improvement, MacTrigger and aPD-1 combination therapy would be a promising approach to overcome the low reactivity of current ICI therapy.

## 4. Conclusions

This study reports a novel methodology that can enhance aPD-1 reactivity in tumor tissues without affecting normal tissues. MacTriggers can induce increased PD-1/PD-L1 expression levels through inflammation in tumor tissues, whereas this was not observed in normal tissues. This tumor-specific upregulation resulted from M2 phenotype inflammation induction, the unique advantage of MacTriggers. Importantly, this PD-1/PD-L1 upregulation does not depend on antigen presence or EPR effects. From these characteristics, this approach could theoretically be applied to almost all tumors. Here, we focused on two tumor types, 4T1 and Colon 26 tumors, because of their known ICI resistance or unmet medical needs. This study showed that MacTriggers could enhance the upregulation of both PD-1 and PD-L1 in tumor tissues, leading to higher aPD-1 efficacy. Additionally, this methodology may also be applied to ICI-resistant tumors with low CD8^+^ T cell levels, despite high initial PD-L1 expression patterns, because MacTriggers can also infiltrate PD-1-positive CD8^+^ T cells into tumor tissues. Our findings propose a novel approach for overcoming the low response rates of current ICIs.

## Data Availability

Data is contained within the article or Supplementary Material.

## Supplementary Materials

The following supporting information can be downloaded at: https://www.mdpi.com/article/10.3390/cancers16223787/s1, Figure S1: Gating strategy of flow cytometry for cancer cells, macrophages, regulatory T cells, and CD8^+^ T cells; Figure S2: Gating strategy of flow cytometry for tumor necrosis factor (TNF)-α^+^ and interferon (IFN)-γ^+^ cells in neutrophils, macrophages, and T cells; Figure S3: Schematic illustration of experiment for investigation of appropriate administration order; Figure S4: Time course of tumor volume measurements over 14 days after administration in combination therapy; Figure S5: Survival rates of mice in each treatment group; Figure S6: Time course of body weight measurements; Figure S7: Percent of liver weight/body weight 14 days after treatment; Figure S8: Percent of spleen weight/body weight 14 days after treatment; Figure S9: Schematic illustration of experiment for investigation of aPD-1 multiple administration; Figure S10: Time course of tumor volume measurements over 12–14 days in 4T1 (i) and Colon-26 (ii) tumor-bearing mice; Figure S11: Time course of body weight measurements.
