# Supplementary material for: Inflammation-Triggering Engineered Macrophages (MacTriggers) Enhance Reactivity of Immune Checkpoint Inhibitor Only in Tumor Tissues
Source: Cancers (Basel). 2024 Nov 10;16(22):3787. doi: 10.3390/cancers16223787 (PMC11592725; doi:10.3390/cancers16223787)
Supplement: Supplementary file 1 [file cancers-16-03787-s001.zip › cancers-3240159-supplementary.pdf]

Supporting information

Materials and Methods

1. Investigation of order of administration

We investigated the impact of the administration order of MacTrigger and anti-programmed cell death-1 antibody (aPD-1) on anti-tumor effects. All animal experiments were performed with approval from the Institutional Animal Care and Use Committee of Kyushu University. Female BALB/cAJcl mice aged 5-7 weeks were obtained from Kyudo Japan (Saga, Japan). After a week of breeding, 4T1 cells (2×10^5 cells in 50 μL HBSS) were subcutaneously (s.c.) injected into the back. Five days after tumor inoculation, we set the three treatment groups: (i) MacTrigger and aPD-1 (1×10^6 cells and 50 μg in 100 μL PBS) at the same time (i.e., MacTrigger+aPD-1), and (ii) MacTrigger (1×10^6 cells in 100 μL PBS) and then aPD-1 (50 μg in 100 μL PBS) 4 d apart (i.e., MacTrigger→aPD-1), (iii) aPD-1 (50 μg in 100 μL PBS) and then MacTrigger (1×10^6 cells in 100 μL PBS) 4 d apart (i.e., aPD-1→MacTrigger). Each group was administered intravenously. Tumor volumes and body weights were measured every 2 days. The tumor volumes were calculated using the following formula.

$$\text{Tumor volume} = 0.5 \times \text{Major axis} \times (\text{Minor axis})^2 \qquad (1)$$

When the tumor major axis reached 20 mm (humane endpoint), the experiment was finished and the mice were sacrificed. The liver and spleen tissues were then extracted. The liver and spleen tissue weights were measured to assess the tissue enlargement associated with injury.

2. Evaluation of anti-tumor effect by multiple administration of aPD-1

The two types of tumor-bearing mice (4T1 and Colon-26) were obtained using the same methods. Five days after tumor inoculation, we set three treatment groups: (i) PBS (100 μL), (ii)(iii) MacTrigger+aPD-1 (1×10^6 cells and 50 μg in 100 μL PBS). Then, 4 and 8 days after administration, aPD-1 (50 μg in 100 μL PBS) was administered in the group (iii). Each group was administered intravenously. Tumor volumes and body weights were measured every 2 days. The tumor volumes were calculated using the formula (1). When the tumor major axis reached 20 mm (humane endpoint), the experiment was finished and the mice were sacrificed.

3. Comparison of adverse effect between chemotherapy and combination therapy with MacTrigger and aPD-1

4T1-bearing mice were obtained using the same methods. Five days after tumor inoculation, we set three treatment groups: (i) PBS (100 μL), (ii) MacTrigger+aPD-1 (1×10^6 cells and 50 μg in 100 μL of PBS), and (iii) Doxorubicin (DOX)+Cyclophosphamide (CPA) (40 μg and 2 mg in 100 μL of PBS). Four days after drugs administration, the same amounts of drugs were administered in the group (iii). Each group was administered intravenously. Body weights were measured every 2 days. When the body weight loss reached 20% (humane endpoint), the mice were sacrificed.

Supporting information

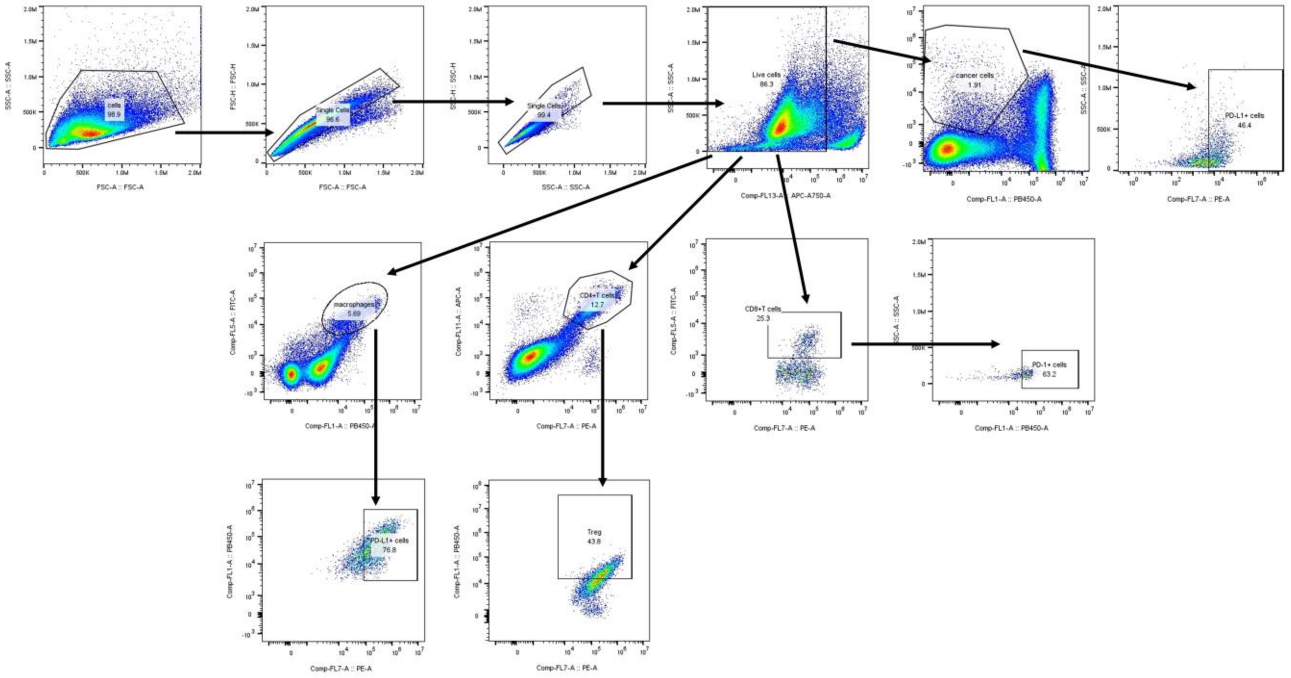

Figure S1. Gating strategy of flow cytometry for cancer cells, macrophages, regulatory T cells, and $CD8^+$ T cells. This gating strategy was used in Fig. 3A, 3B, 3C, and Fig. 5E.

Supporting information

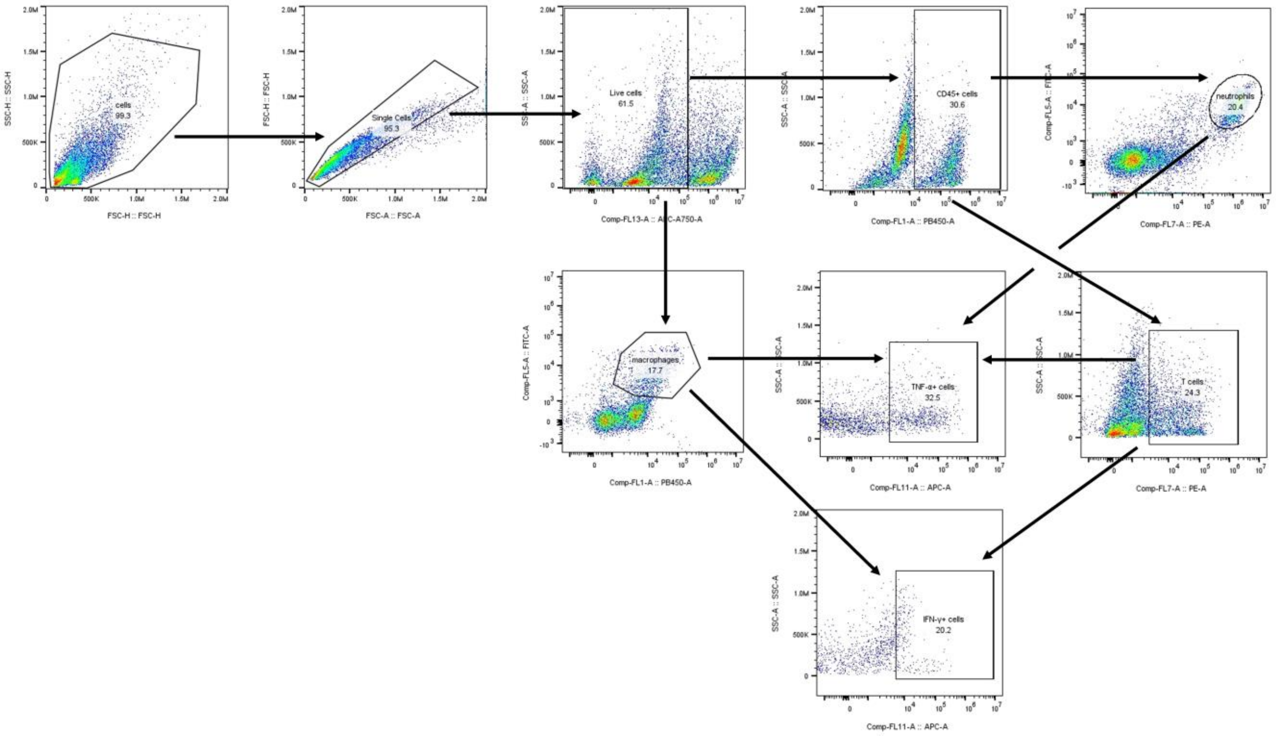

Figure S2. Gating strategy of flow cytometry for tumor necrosis factor (TNF)-α^+ and interferon (IFN)-γ^+ cells in neutrophils, macrophages, and T cells. This gating strategy was used in Fig. 4A, 4B, 4C, 4D, and 4E.

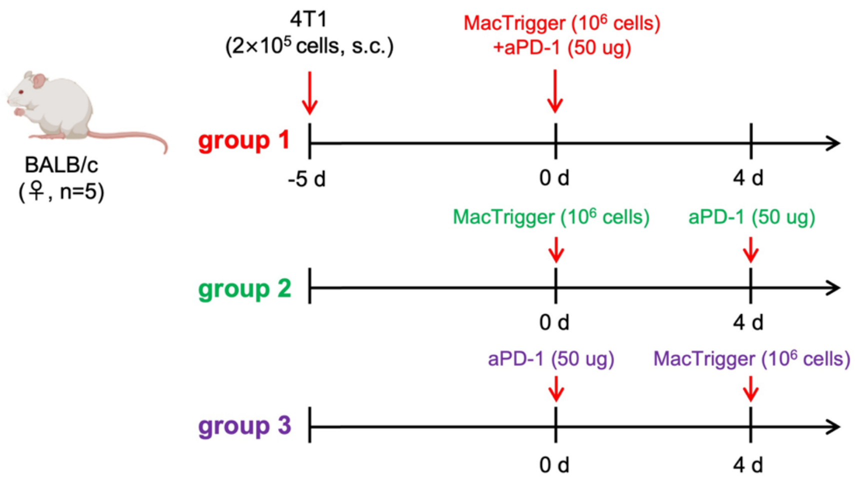

Figure S3. Schematic illustration of experiment for investigation of appropriate administration order.

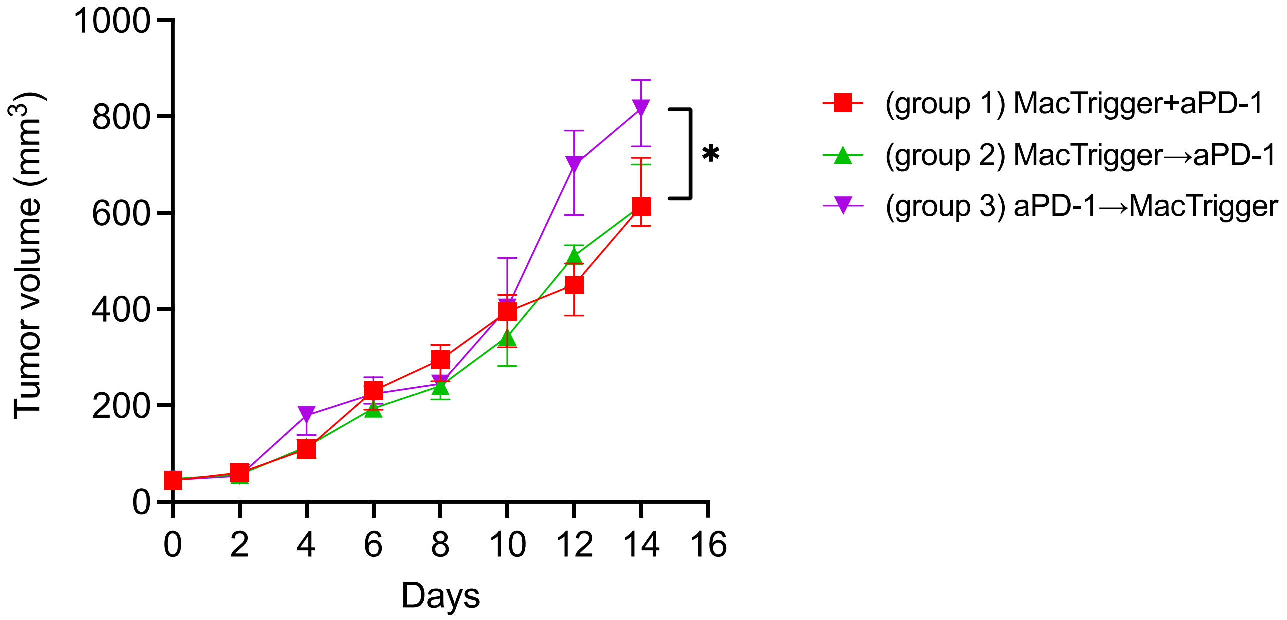

Figure S4. Time course of tumor volume measurements over 14 days after administration in combination therapy (n=5, median (IQR)). $^{*}P < 0.05$.

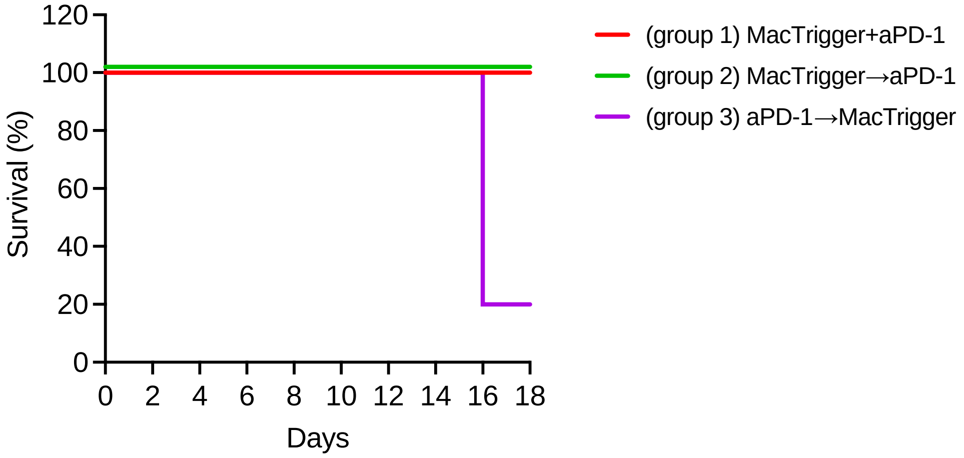

Figure S5. Survival rates of mice in each treatment group (n=5).

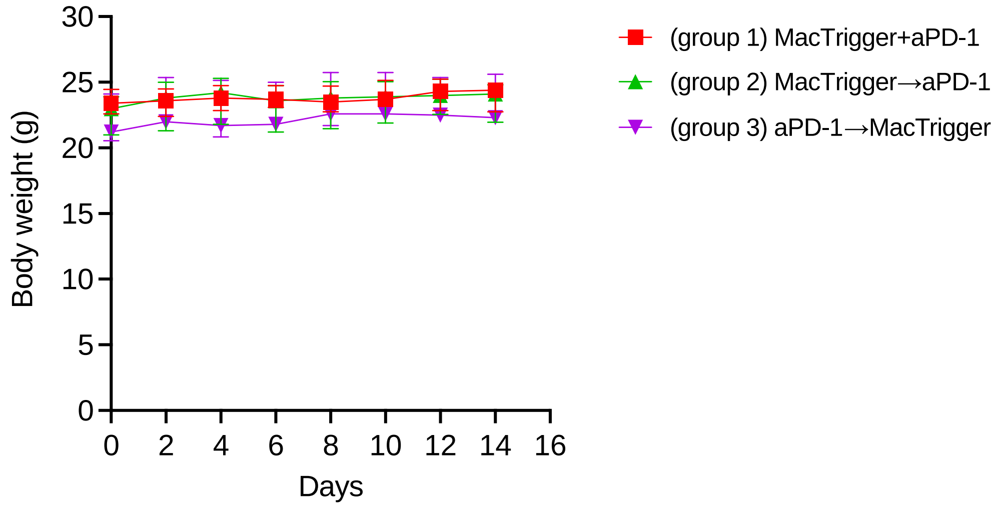

Figure S6. Time course of body weight measurements (n=5, median (IQR)).

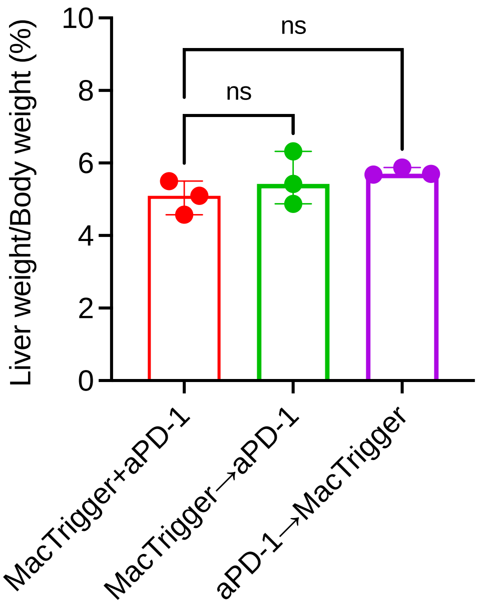

Figure S7. Percent of liver weight/body weight 14 days after treatment (n=3, median (IQR)). ns: not significant.

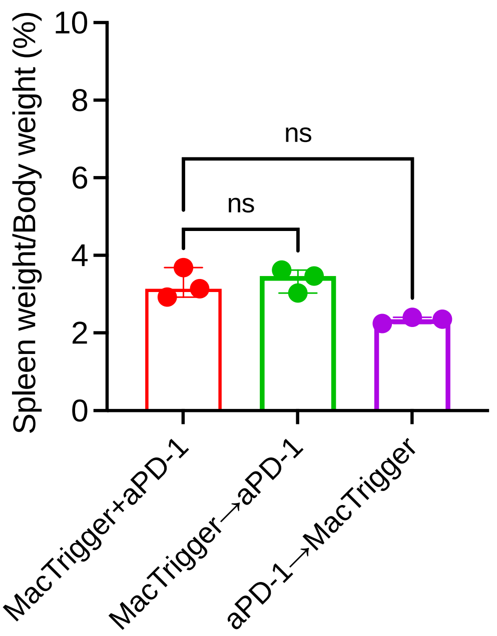

Figure S8. Percent of spleen weight/body weight 14 days after treatment (n=3, median (IQR)). ns: not significant.

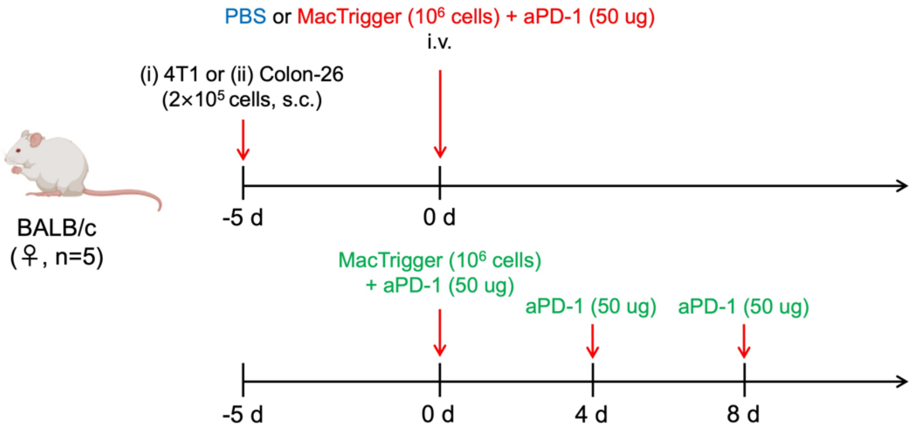

Figure S9. Schematic illustration of experiment for investigation of aPD-1 multiple administration.

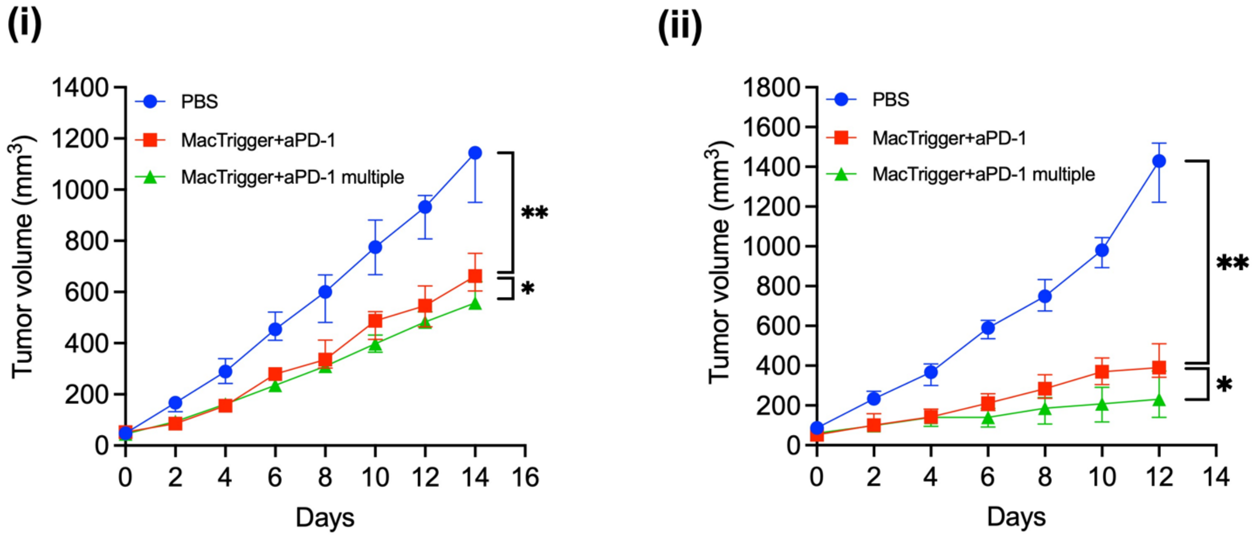

Figure S10. Time course of tumor volume measurements over 12-14 days in 4T1 (i) and Colon-26 (ii) tumor-bearing mice (n=5, median (IQR)). $^{*}P < 0.05$, $^{**}P < 0.01$.

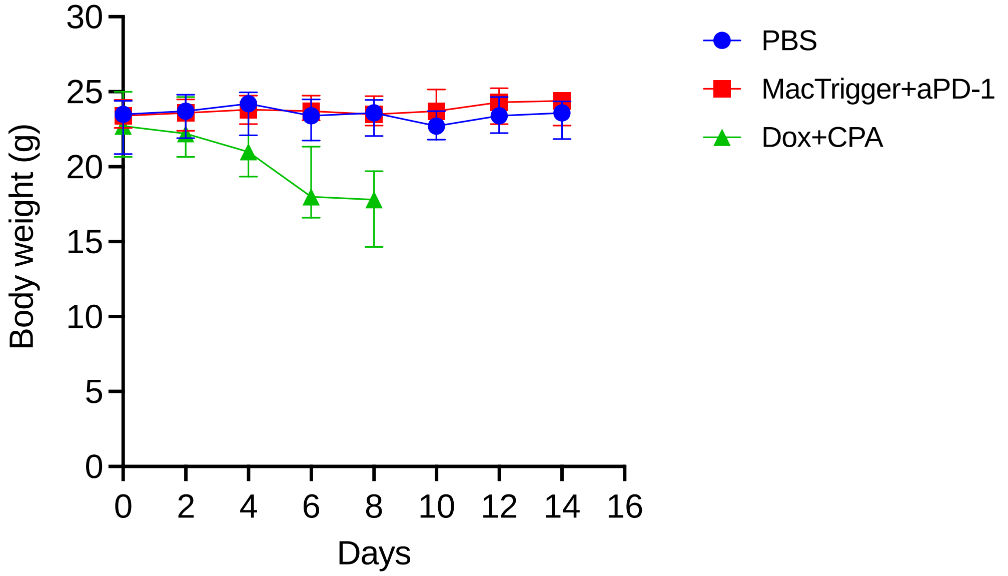

Figure S11. Time course of body weight measurements (n=5, median (IQR)).
